# Exaggerated postnatal surge of orexin neurons and the effects of elimination of excess orexin on blood pressure and exaggerated chemoreflex in spontaneously hypertensive rats

**DOI:** 10.3389/fphys.2024.1341649

**Published:** 2024-10-09

**Authors:** Savannah Lusk, Alexander M. Moushey, Nicholas Iwakoshi, Christopher G. Wilson, Aihua Li, Russell Ray

**Affiliations:** ^1^ Dartmouth College, Department of Molecular and Systems Biology, Hanover, NH, United States; ^2^ Loma Linda University, Center for Perinatal Biology, Loma Linda, CA, United States; ^3^ Baylor College of Medicine, Department of Neuroscience, Houston, TX, United States

**Keywords:** hypertension, orexin, postnatal neurogenesis, hypercapnia, hypercapnic chemoreflex

## Abstract

An overactive orexin (OX) system is associated with neurogenic hypertension and an exaggerated chemoreflex in spontaneously hypertensive rats (SHRs). However, the chronology and mechanism of this association is unclear. We hypothesized that increased postnatal neurogenesis of OX neurons in SHRs precedes and contributes to the aberrant increase in mean arterial blood pressure (MAP) and the exaggerated response to hypercapnia during postnatal development. Using immunohistochemical methods and bromodeoxyuridine, we mapped the timeline of orexin neuron neurogenesis and maturation during early postnatal development. We then used whole-body plethysmography with EEG and EMG to map the development of mean arterial pressure (MAP) and state regulation. Finally, we used OX-targeted saporin toxin to determine the effects of eliminating excess OX neurons on the elevated MAP and exaggerated chemoreflex in adult SHRs. We found that both SHRs and Wistar–Kyoto (WKY) rats experienced postnatal increases in OX neurons. However, SHRs experienced a greater increase than WKY rats before P15, which led to significantly more OX neurons in SHRs than age-matched WKY controls by P15–16 (3,720 ± 780 vs. 2,406 ± 363, *p* = 0.005). We found that neurogenesis, as evidenced by BrdU staining in OX-positive neurons, was the primary contributor to the excess OX neurons in SHRs during early postnatal development. While SHRs develop more OX neurons by P15–16, SHRs and normotensive WKY control rats have similar MAP during postnatal development until P25 in wakefulness (81.6 ± 6.6 vs. 67.5 ± 6.8 mmHg, *p* = 0.006) and sleep (79.3 ± 6.1 vs. 66.6 ± 6.5, *p* = 0.009), about 10 days after the surge of OX neurons. By selectively eliminating excess (∼30%) OX neurons in SHRs, we saw a significantly lowered MAP and hypercapnic ventilatory chemoreflex compared to non–lesioned SHRs at P40. Additionally, we found unique signatures in state indicative of the attention defecit phenotype commonly associated with this model. We suggest that the postnatal increase of OX neurons, primarily attributed to exaggerated postnatal OX neurogenesis, may be necessary for the development of higher MAP and exaggerated chemoreflex in SHRs, and modulation of the overactive OX system may have a potential therapeutic effect during the pre-hypertensive period.

## 1 Introduction

Hypertension is a major health concern associated with increased risks for heart disease, stroke, and other serious health issues while being associated with nearly 700,000 deaths each year in the United States alone (CDC). Hypertension treatments often focus on sodium management and the renin–angiotensin system. However, about 95% of hypertensive cases can be categorized as essential hypertension wherein the disease is idiopathic and no secondary causes of hypertension are present (Carretero and Oparil, 2000). Thus, a better understanding of the circuits and mechanisms underlying neural control of blood pressure and neurogenic hypertension is needed to inform upon patient care.

Participation of hypothalamic orexinergic (OX) neurons in cardiorespiratory regulation has been well established over the last 2 decades (Li and Nattie, 2014; Samson et al., 1999; Shirasaka et al., 1999; Shahid et al., 2011). OX neurons project to and excite many cardiorespiratory control nuclei in the central nervous system, including the paraventricular nucleus (PVN), nucleus tractus solitarius (NTS), retrotrapezoidal nucleus (RTN), medullary raphe, rostro–ventrolateral medulla (RVLM), and sympathetic preganglionic neurons of the intermediolateral column (Peyron et al., 1998; Trivedi et al., 1998; Shirasaka et al., 2001; Marcus et al., 2001; Yang and Ferguson, 2003; Huang et al., 2010; Shahid et al., 2012; Van Den Pol, 1999; Antunes et al., 2001; van den Top et al., 2003). Central administration of OX leads to significant and sustained increases in mean arterial blood pressure (MAP), heart rate, catecholamine release, and renal sympathetic nerve activity (RSNA) in conscious and anesthetized rats, an effect that is prevented by pre–treatment with OX receptor antagonists (Li and Nattie, 2014; Samson et al., 1999; Shirasaka et al., 1999; Shahid et al., 2011; Huang et al., 2010).

A link between an overactive OX system and the pathophysiology of neurogenic hypertension has been established in spontaneously hypertensive rats (SHRs) and mice (BPH/2J) (Jackson et al., 2016; Lee et al., 2015; Lee et al., 2013; Li et al., 2013; Clifford et al., 2015). On average, adult spontaneously hypertensive mice (BPH/2J) and rats have about 30% more OX–producing neurons than their normotensive background controls (Jackson et al., 2016; Lee et al., 2015; Li et al., 2016). Inhibition of OX neurons and their signaling in the hypertensive rat model using a dual OX receptor antagonist can 1) significantly lower MAP (by 20–30 mmHg), sympathetic nerve activity (SNA), and catecholamine release in conscious adult SHRs (Li et al., 2013) and normalize MAP in younger SHRs (postnatal day (P)30–58), and 2) normalize the exaggerated ventilatory hypercapnic chemoreflex at both ages (Li et al., 2016). These findings suggest that excessive OX–producing neurons may play important roles in the pathophysiology of neurogenic hypertension in SHRs including the regulation of both MAP and the hypercapnic ventilatory chemoreflex.

OX neurons proceed through pre–and post–natal developmental changes and mature postnatally in mammals (Yamamoto et al., 2000; Steininger et al., 2004; Stoyanova et al., 2010; Iwasa et al., 2015; Sawai et al., 2010; Amiot et al., 2005). Using *in situ* hybridization, Yamamoto et al. showed that *prepro–orexin* mRNA in the rat hypothalamus gradually increased during early postnatal development and peaked by 3 weeks of age (Yamamoto et al., 2000). Further, Sawai et al. showed a clear increase in the number of OXA–and OXB–peptide–expressing neurons from 2 weeks to 9 weeks of age (Sawai et al., 2010). It is not clear whether the postnatal increase in OX was due to postnatal maturation of existing neurons or neurogenesis. Additionally, these studies were completed in normotensive animals and did not include comprehensive cell counts at various ages.

Currently, little is known about whether the increased number of OX neurons in SHRs is developed prenatally or postnatally, how OX neuron development and blood pressure development are chronologically correlated, and whether or not modulation of the OX system can change the course of developing higher MAP during development.

In this study, using SHRs and their normotensive background control Wistar–Kyoto (WKY) rat pups, we aimed to address the following questions: 1) What is the developmental timeline of MAP and number of OX neurons in SHRs and WKY rats; 2) What is the primary mechanism that produces excess OX–producing neurons during postnatal development in SHRs; 3) What is the chronological relationship between excess OX neurons and the development of hypertension in SHRs and WKYs; and 4) Does elimination of excess OX neurons in SHRs have an impact on the development of a higher MAP and exaggerated hypercapnic chemoreflex during development. We hypothesized that increased postnatal OX neurogenesis leads to a postnatal increase of OX neurons in SHRs, which is required for the aberrant increase in MAP in SHRs, and that elimination of these excess OX neurons can prevent SHRs from developing a higher MAP and exaggerated hypercapnic chemoreflex during early development.

## 2 Methods

### 2.1 Ethical approval

All animal experimental and surgical protocols were within the guidelines of the National Institutes of Health for animal use and care and were approved by the Institutional Animal Care and Use Committee at the Geisel School of Medicine at Dartmouth. All animals were reared and transported in accordance with regulations of these governing bodies.

### 2.2 Animal use and care

Spontaneously hypertensive rats (SHRs, Charles River strain 007) and normotensive Wistar–Kyoto (WKY, Charles River strain 008) rats were used for the experiments in this study. All rats were housed in a temperature–and light–controlled environment set on a 12 h–12h light–dark cycle (lights on at 00.00 h; lights off at 12.00 h). Food and water were provided *ad libitum*. A total of 75 SHRs and 44 WKY rats between the ages of P7 and P40 were used in four sets of anatomical and physiological experiments, and males and females were evenly distributed across each group. At the conclusion of the experiments, the rats were euthanized with an overdose of sodium pentobarbital (>75 mg kg^–1^, i.p.; Euthasol; Virbac Inc., Fort Worth, TX, United States: PVS111).

### 2.3 Surgeries and procedures

*Blood pressure probes*. All animals used to study MAP were surgically implanted with a telemetric blood pressure probe. For animals aged P7–P30, rats were anesthetized with 4% isoflurane (Piramal Enterprises Ltd.: NDC#66794–017) for initial induction followed by 1%–2% isoflurane to maintain a surgical level of anesthesia. Ketoprofen (3 mg/kg, subcutaneously) was used as an analgesic after surgery. Rats were implanted with an HD–X11 telemetric probe (DSI, St. Paul, MN, United States), which allows continuous, reliable recording of MAP and body temperature in wakefulness and sleep as similarly described in previous publications (Li et al., 2013; Li et al., 2016; Li et al., 2008). In brief, the catheter of the probe was inserted into the descending aorta of the rat via the femoral artery for MAP; two wire leads were tunneled subcutaneously and placed on the surface of the parietal bone in the skull for EEG, and the body of the HD–X11 transmitter was placed inside the abdominal cavity for core body temperature. The incision site was sutured, and animal allowed to recover for at least 2 days prior to whole body plethysmography.

For animals aged P35–40, rats were anesthetized with a ketamine (Putney, Inc., Portland, ME, United States: NDC#67457–181) and xylazine (Lloyd Labs, Walnut, CA, United States: NDC#59399–110) cocktail (90/15 mg kg^–1^, I.M.). Ketoprofen (3 mg/kg, subcutaneously) was used as an analgesic after surgery. Rats were implanted with a PA–C10 telemetric probe (DSI, St. Paul, MN, United States), which allows for uninterrupted and reliable recording of MAP during the experiment as similarly described in our previous publications (Li et al., 2013; Li et al., 2016; Li et al., 2008). In brief, the catheter of the probe was inserted into the descending aorta of the rat via the femoral artery and was secured between the descending aorta and the renal artery. The probe was fastened in a subcutaneous pocket in the flank region on the corresponding side of the target femoral artery. Incision sites were sutured, and animals allowed to recover for at least 2 days prior to whole body plethysmography.

*EEG/EMG.* For EEG activity in P7–P30 rats, two wire leads of HD–X11 transmitter were tunneled subcutaneously on to the surface of the parietal bone in the skull. This was performed during the blood pressure probe procedure and, therefore, all drugs used were the same for these animals as those outlined above.

For animals at P35–40, 7 days prior to whole body plethysmography experiments, rats were anesthetized with a ketamine (Putney, Inc., Portland, ME, United States: NDC#67457–181) and xylazine (Lloyd Labs, Walnut, CA, United States: NDC#59399–110) cocktail (90/15 mg kg^–1^, I.M.). Following confirmed anesthesia, three EEG electrodes were screwed onto the skull (from bregma: 1) +2 mm lateral, +2 m rostral; 2) +2 mm lateral, −3 mm caudal; 3) −2 mm lateral, −2 mm caudal) resulting in a fronto–parietal EEG with a group attachment on the opposite hemisphere. In these same P35–40 animals, two EMG electrodes were sutured onto the dorsal neck muscles on each lateral side. All electrode leads (3 EEG and 2 EMG) were inserted into a sterilized six–prong plastic pedestal that was secured to the skull using Ortho Jet Acrylic (LangDental, Wheeling, IL, United States). The incision was sutured and the animal allowed to recover for at least 7 days prior to whole body plethysmography. During all procedures level of anesthesia was verified with a toe pinch prior to initiation and anesthesia level was monitored by watching the respiratory patterns of the animal throughout the procedures.

*BrdU (Bromodeoxyuridine) injection.* Newly proliferated OX neurons in the hypothalamus were marked using BrdU and identified by BrdU and OX–ir double staining. Rat pups were injected with BrdU (i.p. 50 mg kg^–1^ day^–1^ BrdU in saline; Roche Diagnostics, Indianapolis, IN, United States) for 5 consecutive days, i.e., from P5 to P9, P10 to 14, or P15 to P19, respectively (hereafter, the simplified P5–9, P10–14 and P15–19 injected rats terminology will be used). For P5–9, rats were euthanized 3–5 days following the last injection and brain tissue was harvested. For P10–14 and P15–19, rats were euthanized 1 day after the final injection and brain tissue harvested.

*Hcrt–SAP injection.* Elimination of excess OX neurons was achieved using an OX neuron–targeted toxin, Hcrt–SAP (hypocretin–2–saporin, Advanced Targeting Systems, San Diego, CA, United States). Rats were anesthetized with a ketamine (Putney, Inc., Portland, ME, United States) and xylazine (Lloyd Labs, Walnut, CA, United States) cocktail (90/15 mg kg^–1^, I.M.), and then fixed in a Kopf stereotaxic frame. Hcrt–SAP or its control IgG–SAP was unilaterally injected (0.5 μL, 90 ng/μL) into the hypothalamus from bregma: 1.4 mm lateral, 2.3 mm caudal and 8.8 mm below the surface of the skull. All injections were made using a 1 μL Hamilton syringe, and each microinjection lasted at least 5 min (maximum injection flow rate: 0.1 μL/min) and the needle remained in position for another 5 min before removal to prevent reuptake of the drug during removal and ensure release of the drug from the needle. Following syringe removal, the incision was closed, and the animal was allowed to recover. Hcrt–SAP was allowed to lesion for 12–day as other studies have shown significant OX neuron cell loss at this timepoint and it is in line with timelines of other saporin–conjugates (Waite et al., 1994; Mantyh et al., 1997; Gerashchenko et al., 2001; Gerashchenko et al., 2003).

### 2.4 Whole body plethysmography

The methods used to measure MAP and EEG were those in common use in our laboratory (Li et al., 2013; Li et al., 2016; Penatti et al., 2011; Cummings et al., 2011). For all animals, body temperature measurements were used in combination with the plethysmography recordings to calculate an estimate of tidal volume and EEG/EMG was used to determine state. EEG/EMG values are not shown as independent findings as they are incorporated in the outcomes presented, i.e., quiet wakefulness *versus* NREM sleep.

*For study in P15–P30 rats*, pups were placed in a water–jacketed glass chamber with body temperature held at 36°C ± 0.5°C throughout the experiment by controlling the temperature of the water perfused through the chamber. The signals of MAP, EEG, core body temperature, and barometric pressure of the HD–X11 telemetric probe were collected continuously throughout the experiment via a PhysioTel Connect device enabler system (DSI, St. Paul, MN, United States) using LabChart 8 software (ADInstruments, Colorado Springs, CO, United States). MAP, systolic pressure, and diastolic pressure signals were sampled at 1,000 Hz. Breathing, EEG, and body temperature were sampled at 150 Hz.

*For study in P37–40 rats*, animals were placed in a traditional whole–body plethysmograph with food ad libidum as described previously (Li et al., 2013; Li et al., 2016). Rectal body temperature was measured before and after recording and those two values were averaged for respiratory calculations. Raw EEG and EMG outputs from the skull and neck skeletal muscle electrodes were filtered at 0.3–70 and 0.1–100 Hz, respectively, using a Grass Physiodata Amplifier System (NatusNeurology Inc., Grass Products, Middleton, WI, United States). All data were collected continuously throughout the experiment via a PhysioTel Connect device enabler system (DSI, St. Paul, MN, United States) using LabChart 8 software (ADInstruments, Colorado Springs, CO, United States). MAP signals were sampled at 1,000 Hz whereas breathing, EEG, and body temperature were sampled at 150 Hz.

### 2.5 Histology

*Tissue processing and harvesting.* Rats were euthanized with an overdose of sodium pentobarbital (>75 mg kg^–1^, i.p.; Euthasol; Virbac Inc., Fort Worth, TX, United States: PVS111) and then transcardially perfused with saline followed by chilled 4% paraformaldehyde (PFA, 4% in 0.1 M phosphate buffer, pH 7.4). The brain was harvested and post–fixed overnight in 4% PFA at 4°C, then cryoprotected in 30% sucrose for at least 48 h at 4°C. 40 *μ*m thick brain sections spanning the entire orexinergic population within the hypothalamus were used for immunohistochemical (IHC) staining of either OX only, or OX and BrdU.

*OX only staining.* Free–floating sections were incubated in anti–orexin–A primary antibody (1:10,000 dilution; goat polyclonal, SC–8070, Santa Cruz, Dallas, TX, United States) for 48 h at 4°C. Sections were washed 3 times for 15 min each with 0.1% Triton X–100 phosphate–buffered saline (PBST) followed by biotinylated horse anti–goat IgG secondary antibody (1:1,00 dilution; Vector laboratories, Burlingame, CA, United States) overnight at 4°C or 2 h at room temperature. Sections were washed 3 times for 15 min each with PBST followed by visualization with peroxidase and diaminobenzidine (DAB) with nickel (black coloring). The visualization reaction was stopped when the color change was visible with the addition of PBST to the wells.

*BrdU and OX double staining*. The brain sections were first denatured with 2N HCl for 45 min, followed by neutralization in 0.1M boric buffer for 30 min (min), and then washed 3 times for 15 min each with PBST. The initial denaturing step allowed for access to the nucleus by the antibodies. The sections were then incubated in mouse anti–BrdU primary antibody (1:100 dilution; G3G4, Developmental Studies Hybridoma Bank, Iowa City, Iowa, United States) for 48 h at 4°C followed by 3 washes for 15 min each with PBST. Sections were then incubated in a biotinylated horse anti–mouse IgG secondary antibody (1:1,000 dilution; Vector Laboratories, Burlingame, CA, United States) overnight at 4°C or 2 h at room temperature. Peroxidase and diaminobenzidine with nickel were used to visualize the BrdU (black nuclei). Brain sections were washed 3 times for 15 min each with PBST, then incubated in a goat polyclonal anti–OXA primary antibody (1:10,000 dilution; SC–8070, Santa Cruz, Dallas, TX, United States) for 48 h at 4°C. Sections were washed 3 times for 15 min each with PBST, then incubated with biotinylated horse anti–goat IgG secondary antibody (1:1,000 dilution; Vector Laboratories, Burlingame, CA, United States) overnight at 4°C or 2 h at room temperature. Peroxidase and diaminobenzidine without nickel were used to visualize OX (brown neurons). The visualization reactions were stopped when the color change was visible with the addition of PBST to the wells.

Three types of neurons were identified for the quantification in this study (Figures 2B,C). BrdU/OX–ir neurons, positive for both BrdU and OX, were identified as brown neurons with black nuclei, which were likely postnatally proliferated OX neurons. OX–ir only neurons, positive for OX–ir but negative for BrdU, were identified as brown without black nuclei, which were generated prior to the postnatal period in this study. BrdU–ir only neurons, positive for BrdU but negative for OX–ir, identified as black nuclei only, which were likely postnatally generated non–OX neurons in the hypothalamus.

*Cell counts.* The hypothalamus was divided into three zones (the dorsomedial hypothalamus (DMH), the perifornical hypothalamus (PeF) and the lateral hypothalamus (LHA)) using count boxes in Neurolucida and Stereo Investigator (MFB Bioscience, Williston, VT, United States). Region boundaries per hemisphere were abut with the PeF centered on the fornix, which was clearly visible in all slices. The angle and size of the regions were changed per hemisphere as needed for each slice to ensure all OX neurons were counted in each study and assigned the proper region. Cells were counted from the live bright field images on the computer software and the microscope view was consulted for any neurons where parfocality was questioned. Cell counts were quantified in total and by hemisphere boxes when the entire slice was completed. There were no z–plane guard zones, but only wholly visible neurons were counted to offset the possibility of double counting neurons that were on the z extremities. That said, all slices were counted this way to ensure consistency across counted brains. For OX only neuron counts, all whole OX–ir neurons were counted. For BrdU and OX neuron counts, all whole OX–only–ir neurons were counted (brown neurons) and all whole BrdU+/OX + neurons were counted (brown neurons with black nuclei). The same investigator completed all cell counts for consistency in identification.

### 2.6 Experimental designs and data analysis

**Experiment 1**: Postnatal developmental changes in number of orexin neurons in SHRs vs. WKYs.

To determine at which developmental age the number of OX–ir neurons in SHRs diverges from normotensive WKY rats, SHR and WKY rat pups were divided into three age groups for brain harvest and OX–ir cell counts, P7–8 (SHR n = 6; WKY n = 3), P15–16 (SHR n = 11; WKY n = 4), and P25–40 (SHR n = 5; WKY n = 6).

*Data analysis:* The total number of OX–ir neurons were quantified and compared between SHRs and WKY rats at three ages (P7–8, P15–17, and P25–40) using a linear mixed effects model with a Tukey posthoc where age, strain (SHR and WKY rats), and region were used as independent variables. All values are reported as mean ± standard deviation.

**Experiment 2**: Postnatal neurogenesis of orexin neurons.

To determine whether SHR pups have greater postnatal proliferation of OX neurons than age matched normotensive WKY pups during development, we used bromodeoxyuridine (BrdU) to mark newly proliferated cells. Rats were divided into three sex–matched age groups P5–9 (SHR n = 5; WKY n = 5), P10–14 (SHR n = 5; WKY rats n = 5), and P15–19 (SHR n = 4; WKY rats n = 3). The brains were harvested 1 day after the fifth BrdU injection except for P5–9 injected rats, which were harvested 3–5 days after the conclusion of BrdU injections. The delay in the P5–9 age group was to make the tissue more resilient to the staining procedure, which tended to be too harsh for the tissue to remain intact if harvested at P10.

*Data analysis:* All OX–ir neurons that were positive for BrdU (BrdU/OX–ir) in the hypothalamus and all OX–ir only neurons were quantified and compared between SHRs and WKY rats at three age groups using a linear mixed effects model with a Tukey posthoc where age, strain (SHR and WKY rats), and region were the independent variables. All values are reported as mean ± standard deviation.

**Experiment 3**: Development of higher MAP in SHRs vs. WKYs.

To determine at which age SHRs develop a significantly higher MAP than age matched normotensive WKY controls and its chronological relationship to postnatal changes in the OX system. Rat pups at four ages were used to measure the developmental change of MAP, P15 (SHR n = 5; WKY n = 4), P20 (SHR n = 9; WKY n = 6), P25 (SHR n = 8; WKY n = 8), and P30 (SHR n = 11; WKY n = 8). Table 1 shows average weights for all rats by strain at each age group and Table 2 show the number of rats, and breakdown of males vs. females measured at each age. One day after implantation of BP telemetry and EEG, rat pups were acclimatized in a water–jacketed chamber for at least 1 h and body temperature was maintained at 36°C ± 0.5°C. MAP, EEG, and body temperature in wakefulness and sleep were collected continuously for 1.five to three h in room air conditions.

**TABLE 1:** Body weight of animals from experiment 3, developmental timeline of blood pressure. Average body weight at P15, P20, P25 and P30 of SHR and WKY rat pups used in Experiment 3. Both SHR and WKY rat pups showed an age–dependent increase in body weight. Data are shown as mean ± standard deviation.

Strain	P15 (g)	P20 (g)	P25 (g)	P30
SHR	22.91 ± 1.6	36.5 ± 9.2	41.8 ± 8.9	63.9 ± 7.2 g
WKY	33.8 ± 3.9	31.8 ± 2.3	40.2 ± 7.9	57.3 ± 9.9

*Data Analysis:* The mean MAP, diastolic blood pressure (DBP) and systolic blood pressure (SBP) of SHR and WKY rat pups in wakefulness and NREM sleep at P15, P20, P25, and P30 were analyzed and compared separately using a linear mixed effects model with a Tukey post–hoc where age and strain (SHR and WKY rats) were independent variables. Body weight was analyzed separately with strain and age as independent variables. All values are reported as mean ± standard deviation.

**Experiment 4**: The effects of eliminating excess orexin neurons on MAP and ventilatory chemoreflex.

To determine if eliminating excess OX neurons via Hcrt–SAP can prevent SHRs from developing a higher MAP and exaggerated hypercapnic response, P30–40 SHRs received either Hcrt or IgG unilateral injections into the perifornical zone of the hypothalamus. SHRs were randomly assigned into two groups to receive either Hcrt–SAP or IgG–SAP injection between P25–28 (n = 6, weight = 90.4 g; n = 5, weight = 82.2 g, respectively). Five days post–injection, rats were implanted with EEG/EMG and 10 days post–injection rats were implanted with blood pressure telemeters. Twelve days post–injection at P37–40, SHRs were allowed to acclimatize for 1–2 h in an experimental whole–body plethysmography. MAP and ventilatory data in wakefulness and sleep were collected for 1–2 h during the dark period in room air and 1–2 h during the dark period in a 5% CO_2_ mixed gas (5% CO_2_ 21% O_2_ balanced with nitrogen). At the conclusion of the recording, the brain was harvested for OX–ir staining and quantification. Ages of both groups at the day of injection and at the day of physiological experiments are included in Table 3.

**TABLE 2:** Number of animals and number of female rats from experiment 3, developmental timeline of blood pressure. Number of rats measured (number of female rats, number of male rats) at P15, P20, P25 and P30 of SHR and WKY rat pups used in Experiment 3. Male and female rats were evenly distributed across all groups.

Strain	P15	P20	P25	P30
SHR	5 (2F, 3M)	9 (4F, 5M)	8 (4F, 4M)	11 (6F, 5M)
WKY	4 (2F, 2M)	6 (3F, 3M)	8 (3F, 5M)	8 (4F, 4M)

**TABLE 3:** Age and body weight of SHRs used in experiment 4, effect on blood pressure of elimination of OX neurons with Hcrt–SAP. There were no statistically significant differences in body weight or age at the day of injection or the day of physiological measurement between Hcrt–SAP and IgG–SAP groups. Data are shown as mean ± standard deviation. Abbreviations: BW, body weight.

Treatment group	Number of animals (# female)	Age at injection	BW at injection (g)	Age at physiology experiment	BW at physiology experiment (g)
Hcrt–SAP	6 (3)	26.2 ± 0.5	56.1 ± 2.6	38.2 ± 0.5	90.4 ± 4.4
IgG–SAP	5 (2)	27.2 ± 0.6	52.7 ± 3.6	39.2 ± 0.6	82.2 ± 5.8

*Data Analysis:* The number of OX neurons, mean MAP and ventilation were compared between Hcrt–SAP and IgG–SAP treated SHRs 12 days post injection. The total number of OX neurons per hemisphere was quantified and tested using a linear mixed effects model with a Tukey post–hoc where hemisphere, region, and treatment (IgG–SAP vs. Hcrt–SAP) were independent variables. We also analyzed the percentage loss of orexin neurons within the injected hemisphere in each animal using the following equation:

Equation 1:

$$Percent\;loss\;of\;OX\;neurons=\left(\frac{\left(\#OX\;neurons\;in\;uninjected\;hemisphere-\#OX\;neurons\;in\;injected\;hemisphere\right)}{\#OX\;neurons\;in\;uninjected\;hemisphere}\right)x\;100$$ (1)

We tested differences in percentage loss of OX neurons using a linear mixed effects model with a Tukey post–hoc where region and treatment (IgG–SAP vs. Hcrt–SAP) were independent variables. All values are reported as mean ± standard deviation.

For the physiological effects of OX lesion, mean MAP, ventilation, respiratory frequency, and tidal volume were compared between Hcrt–SAP and IgG–SAP treated animals in room air and 5% CO_2_ using a linear mixed effects model with a Tukey post–hoc where treatment (IgG–SAP vs. Hcrt–SAP), state (quiet wakefulness vs. NREM sleep), and inspired gas (room air vs. 5% CO_2_) were independent variables. Body weight at injection and physiology were analyzed with a linear mixed effects model with a Tukey post–hoc where treatment and timepoint were independent variables. Age at injection and physiology were analyzed with a linear mixed effects model with a Tukey post–hoc where treatment and timepoint were independent variables. All values are reported as mean ± standard deviation.

We also analyzed the frequency distributions for all EEG signals to determine the effects of lesion and gas on CNS state. We exported two sections of EEG data as text files from the original *LabChart* file for all but one animal. Two 45-min segments of the recording were taken from both room–air following acclimation and from 5% CO_2_ exposure when the gas concentration reached 4%. Each text file was imported into a *Jupyter* notebook running *Python* 3.9.16. Within the *Jupyter* notebook, the EEG file was visualized as a raw time–series, as a Fast Fourier Transform (FFT), and as a Welch periodogram (power spectral density). One animal was removed as an outlier based on these data. We also generated more granular time series spectrograms demonstrating 2.5 min before an EMG spike and 2.5 min after, or 5 min in a quiet EMG period for each group both in room air and 5% CO_2_. Power Spectral Density (PSD) measurements were collected using the FFT results and previous work (Aydin–Abidin et al., 2011; Schneider et al., 2014; Kent et al., 2018) computing bands for delta (0–4 Hz), theta (four to eight Hz), alpha (8–15 Hz), beta (15–30 Hz), and gamma (30–200 Hz) frequencies using the trapezoid rule for integration. All data were combined and normalized values for each band were calculated as a percentage of the total PSD. We checked normality using a Shapiro–Wilk test and then used non–parametric tests to compare the groups (Kruskal–Wallis and Mann–Whitney–U) in *R* 4.2.2.

Data are reported as significantly different with *p*-values less than 0.05 and as a trend with *p*-values between 0.05 and 0.08.

## 3 Results

### 3.1 Postnatal developmental changes in the number of orexin neurons in SHRs *versus* WKYs

**FIGURE 1:**
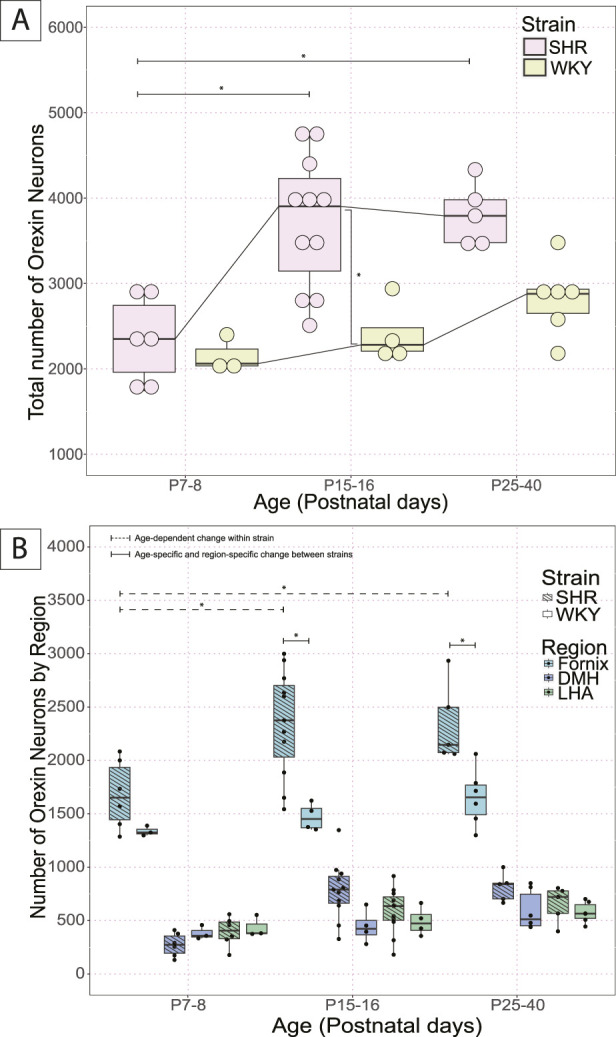
Postnatal developmental changes of OX neurons in the hypothalamus in SHRs and WKY rats. Total number of OX neurons in **(A)** total hypothalamus at three developmental ages, P7–8, P15–16, and P25–40 in SHRs and WKY rats. Where in A, *With a dashed line indicates significant age–dependent differences within a strain, and *With a solid line indicates significant age–specific, region–specific changes between strains. In B, *indicates specific differences and lines indicate between which strains and ages, and **(B)** the three hypothalamic zones (DMH, LHA, PeF). All significant differences were determined as per a linear mixed effects model with a Tukey Honestly Significant Difference (HSD) post–hoc test. Abbreviations: DMH, dorsomedial hypothalamus; PeF, perifornical zone; LHA, lateral hypothalamic area. Data are shown as mean ± standard deviation.

The number of OX–ir neurons in the three hypothalamic zones (dorsomedial hypothalamus: DMH, perifornical zone: PeF, and lateral hypothalamic area: LHA) and across the hypothalamus (three zones combined) at three developmental ages (P7–8, P15–16 and P25–40) are summarized and shown in Figure 1. Only SHRs experienced a developmental increase in the number of OX–ir neurons in the hypothalamus (Figure 1). SHRs showed significantly more OX neurons than WKYs in the total hypothalamus at P15–16 (Figure 1A) but showed significantly more OX neurons specifically in the PeF region at P15–16 and P25–40 (Figure 1B).

SHR pups at ages P15–16 (3720.5 ± 780.3) and P25–40 (3808.4 ± 365.8) had significantly more OX–ir neurons in the total hypothalamus than the pups at P7–8 (2347.2 ± 500.1) (*p* = 0.0006, P7–8 vs. P15–16; *p* = 0.002, P7–8 vs. P25–40; Figure 1A). The increase in total number of OX neurons in the total hypothalamus was primarily contributed by the PeF, where significantly more OX–ir neurons were found in the PeF of pups at P15–16 (2349.5 ± 498.2) and P25–40 (2342.8 ± 375.7) than at P7–8 (1679.8 ± 320.2) (*p* = 0.0003, P7–8 vs. P15–16; *p* = 0.007, P7–8 vs. P25–40; Figure 1B).

In WKY rats, the total number of OX–ir neurons in the hypothalamus increased with age; however, the change was not statistically significant among the three age groups (Figure 1A). There were also no significant differences between specific hypothalamic regions over time in WKY rat pups (Figure 1B).

Between SHR and WKY pups, there was an age-related difference in the number of OX–ir neurons in the total hypothalamus during postnatal development at P15–P16 (SHR = 3720.5 ± 780.3; WKY = 2406.8 ± 363.6) (*p* = 0.005; Figure 1A). At P7–8, there was no statistical difference in the number of OX–ir neurons in the total hypothalamus (SHR = 2347.2 ± 500.1; WKY = 2156 ± 213) or any of the three hypothalamic zones (DMH, PeF, and LHA) between SHR and WKY rat pups (Figure 1A). There was a trend for an increased total number of OX–ir neurons at P25–40 in SHRs compared to WKY rats (SHR = 3808.4 ± 365.8; WKY = 2823.5 ± 429.1) (*p* = 0.073; Figure 1A).

Additionally, no statistically significant differences were noted between SHRs and WKY rats in the DMH or LHA at any age. However, there was a statistically significant difference in the PeF between SHRs and WKY rats at P15–16 (SHR = 2349.5 ± 498.2; WKY = 1470.3 ± 127.9; *p* = 1.54 × 10^−5^) and P25–40 (SHR = 2342.8 ± 375.7; WKY = 1652.3 ± 266.8; *p* = 0.004) (Figure 1B).

### 3.2 Postnatal neurogenesis of orexin neurons

**FIGURE 2:**
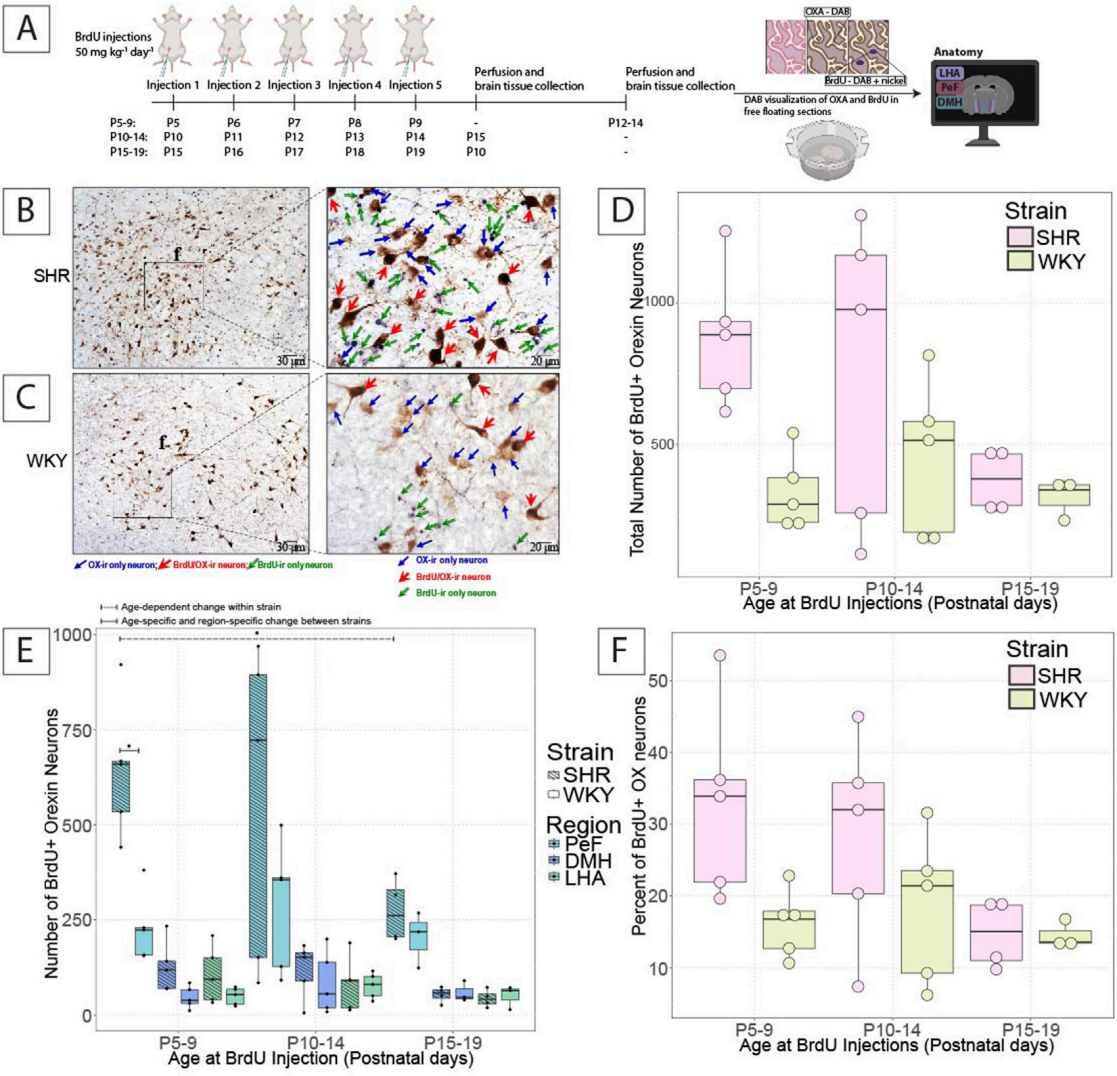
Postnatal OX neurogenesis in SHRs and WKY rats at three developmental periods. **(A)** Shows the experimental paradigm for the BrdU experiments shown. Representative images show OX–ir and BrdU/OX–ir neurons within comparable hemispheres of the hypothalamus in **(B)** SHRs and **(C)** WKY rats from the P5–9 group, and an expanded view of OX–ir neurons (blue arrows; brown neurons), BrdU/OX–ir neurons (red arrows; brown neurons with black nuclei), and BrdU–ir nuclei (green arrows; black nuclei). The total number of BrdU/OX–ir neurons in **(D)** the hypothalamus and **(E)** the three hypothalamic zones in SHRs and WKY rats in each BrdU–injected age group are shown. Where in E, *With a dashed line indicates significant age–dependent differences within a strain, and *With a solid line indicates significant age–specific, region–specific changes between strains. **(F)** Shows the percentage of OX neurons positive for BrdU. All statistical differences were determined as per a linear mixed effects model with a Tukey HSD post–hoc test. Abbreviations: DMH, dorsomedial hypothalamus; PeF, perifornical zone; LHA, lateral hypothalamic area; BrdU, bromodeoxyuridine; OX, orexin–A; ir, immunoreactive. Data are shown as mean ± standard deviation.

A schematic of the BrdU injection, tissue harvesting, and staining protocol is shown in Figure 2A. Representative images of newly proliferated OX–ir neurons marked by BrdU are shown for SHRs (Figure 2B) and WKY rats (Figure 2C).

In SHRs, there was a non-significant age-dependent–reduction in the number of newly proliferated BrdU/OX–ir neurons in the hypothalamus at P5–9 (878.2 ± 248.2), P10–14 (765 ± 546.3), and P15–19 (372 ± 111.7) (Figure 2D). The majority of BrdU/OX–ir neurons were found in the PeF, where significantly more BrdU/OX–ir neurons were observed in P5–9 (644.4 ± 180.8) injected pups than in P15–19 injected pups (274 ± 84), (*p* = 0.012; Figure 2E). No statistically significant age-dependent changes in BrdU/OX–ir neurons in SHRs in the DMH or LHA or the percentage of OX–ir neurons positive for BrdU were found (Figure 2F).

In WKY rats, there were no age-dependent statistically significant changes in the number of BrdU/OX–ir neurons in the total hypothalamus at any age tested. There were also no statistically significant changes in the number of BrdU/OX–ir neurons in WKY rats within any of the three hypothalamic regions between the three ages studied. We also found no significant differences in the percentage of OX–ir neurons positive for BrdU in WKY rats (Figure 2F).

When comparing the difference between SHR and WKY rat pups, there was a trend for more BrdU/OX–ir neurons in the total hypothalamus in SHRs compared to WKY rats at P5–9, but not P10-14 (SHR = 878.2 ± 248.2; WKY = 329.6 ± 134.9) (*p* = 0.079, P5–9; Figure 2D). There were no significant differences in the percentage of OX–ir neurons positive for BrdU at P5–9 and P10–14 (Figure 2F). There was a significant difference in the hypothalamic PeF between SHRs, and WKY rats, where P5–9 (644.4 ± 180.8) injected SHR pups had significantly more BrdU/OX–ir neurons than that of age-matched WKY rat pups (229.8 ± 91.5), (*p* = 0.00096; Figure 2E). There were no significant differences in the DMH or LHA between SHRs and WKY rats at any age studied.

### 3.3 Development of higher MAP in SHRs vs. WKY rats

**FIGURE 3:**
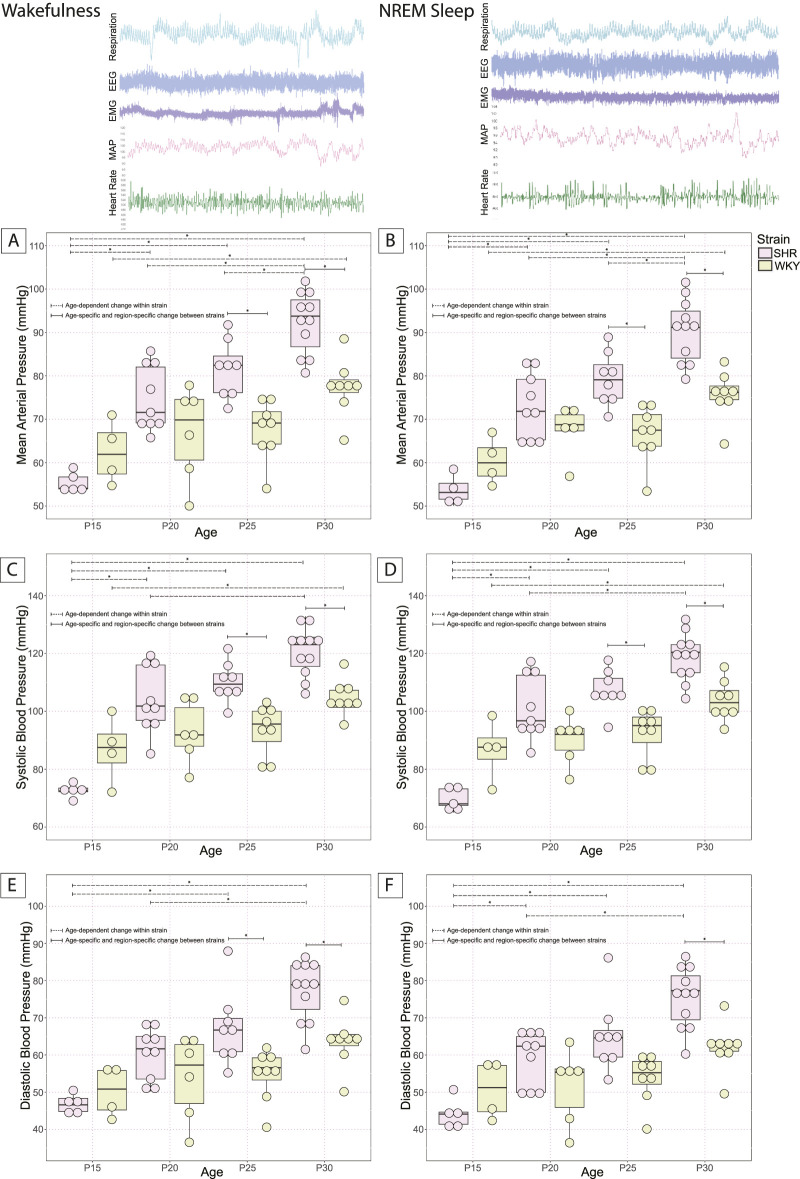
Postnatal changes of MAP in SHRs and WKY rats. MAP at P15, P20, P25, and P30 in SHR and normotensive WKY rats in **(A)** wakefulness and **(B)** NREM sleep are shown. Systolic pressure in SHRs and WKY rats is shown in **(C)** wakefulness and **(D)** NREM sleep. Diastolic pressure in SHRs and WKY rats is shown in **(E)** wakefulness and **(F)** NREM sleep. In all panels, *With a dashed line indicates significant age–dependent differences within a strain, and *With a solid line indicates significant age–specific, region–specific changes between strains. All significant differences were determined using a linear mixed effects model with a Tukey HSD post–hoc test. Data are shown as mean ± standard deviation.

Developmental changes of MAP were accessed in SHRs and their WKY normotensive controls at P15, P20, P25, and P30 to outline the timeline for the development of a higher MAP and ultimately compare this timeline with the development of excess OX–ir neurons (Figure 3). The conditions between age-matched SHRs and their background normotensive control WKY pups are similar. There were age-related increases in body weight in both SHRs and WKY rats. However, there were no significant differences in body weight between the two strains at any age (Table 1). Mean MAP, SBP, and DBP of SHRs and WKY rat pups at P15, P20, P25, and P30 are shown in wakefulness (Figures 3A,C,E) and NREM sleep (Figures 3B,D,F).

Both SHRs and WKY rats experienced postnatal developmental increases in blood pressure. In SHR pups, an age-dependent increase in MAP was observed in both wakefulness and sleep between the age groups P15 (55.4 ± 2.3 and 52.7 ± 3.9 mmHg, respectively), P20 (74.8 ± 7.5 and 73.1 ± 7.5 mmHg, respectively), P25 (81.6 ± 6.6 and 79.3 ± 6.1 mmHg, respectively), and P30 (92.3 ± 7.1 and 90.4 ± 7.2 mmHg, respectively) [Wakefulness: P15 vs. P20 (*p* = 0.0003), P25 (*p* = 1.5 × 10^−6^), P30 (*p* = 1.7 × 10^−11^); P20 vs. P30 (*p* = 4.6 × 10^−5^); P25 vs. P30 (*p* = 0.047); Figure 3A] [NREM Sleep: P15 vs. P20 (*p* = 3.9 × 10^−5^), P25 (*p* = 1.8 × 10^−7^), P30 (*p* = 0); P20 vs. P30 (*p* = 1.4 × 10^−5^); P25 vs. P30 (*p* = 0.017); Figure 3B].

An age-dependent increase in SBP was also observed in SHRs in both wakefulness and sleep between the age groups P15 (72.5 ± 2.4 and 69.6 ± 3.9 mmHg, respectively), P20 (103.9 ± 11.5 and 101.2 ± 11.1 mmHg, respectively), P25 (110.2 ± 6.7 and 107.3 ± 6.95 mmHg, respectively), and P30 (120.6 ± 8.4 and 118.7 ± 8.2 mmHg, respectively) [Wakefulness: P15 vs. P20 (*p* = 1.1 × 10^−6^), P25 (*p* = 1.8 × 10^−8^), P30 (*p* = 0); P20 vs. P30 (*p* = 0.002); Figure 3C] [NREM Sleep: P15 vs. P20 (*p* = 3.1 × 10^−7^), P25 (*p* = 5.3 × 10^−9^), P30 (*p* = 0); P20 vs. P30 (*p* = 0.00058); Figure 3D].

An age-dependent increase in DBP was also observed in SHRs in both wakefulness and sleep between the age groups P15 (46.9 ± 2.6 and 44.2 ± 4.0 mmHg, respectively), P20 (60.3 ± 6.9 and 59.0 ± 7.3 mmHg, respectively), P25 (67.3 ± 9.9 and 65.3 ± 9.8 mmHg, respectively), and P30 (77.3 ± 8.1 and 75.5 ± 8.2 mmHg, respectively) [Wakefulness: P15 vs. P25 (*p* = 0.0009), P30 (*p* = 8.98 × 10^−8^); P20 vs. P30 (*p* = 0.0004); Figure 3E] [NREM Sleep: P15 vs. P20 (*p* = 0.027), P25 (*p* = 0.0004), P30 (*p* = 3.2 × 10^−8^); P20 vs. P30 (*p* = 0.0005); Figure 3F].

In normotensive WKY rat pups, MAP also rose with age during wakefulness and sleep between the age groups P15 (62.4 ± 7.3 and 60.4 ± 5.4, respectively) and P30 (77.4 ± 6.5 and 75.6 ± 5.5, respectively) [Wakefulness: P15 vs. P30 (*p* = 0.03); Figure 3A] [NREM sleep: P15 vs. P30 (*p* = 0.01); Figure 3B]. SBP also rose with age in WKY rats during wakefulness and sleep between the age groups P15 (86.8 ± 11.6 and 86.7 ± 10.5, respectively) and P30 (104.5 ± 6.3 and 103.7 ± 6.9, respectively) [Wakefulness: P15 vs. P30 (*p* = 0.03); Figure 3C] [NREM sleep: P15 vs. P30 (*p* = 0.03); Figure 3D]. There were no statistically significant changes in diastolic blood pressure with age in WKY rats.

Between SHR and WKY rat pups, there was an age-dependent difference in MAP during the developmental period studied. At P25, SHRs showed a significantly higher MAP than WKY rats in wakefulness (SHR = 81.6 ± 6.6; WKY = 67.5 ± 6.8; *p* = 0.006; Figure 3A) and in sleep (SHR = 79.3 ± 6.1; WKY = 66.6 ± 6.5; *p* = 0.009; Figure 3B). We saw the same significant difference in MAP at P30 in wakefulness (SHR = 92.3 ± 7.1; WKY = 77.4 ± 6.5; *p* = 0.001; Figure 3A) and sleep (SHR = 90.4 ± 7.2; WKY = 75.6 ± 5.5; *p* = 0.0004; Figure 3B). We found a significant difference in SBP between SHRs and WKY rats at P25 both in wakefulness (SHR = 110.2 ± 6.7; WKY = 93.6 ± 8.6; *p* = 0.009; Figure 3C) and sleep (SHR = 107.3 ± 7.0; WKY = 92.5 ± 8.3; *p* = 0.02; Figure 3D). We also found a significant difference in SBP between SHRs and WKY rats at P30 in wakefulness (SHR = 120.6 ± 8.4; WKY = 104.5 ± 6.3; *p* = 0.005; Figure 3C) and sleep (SHR = 118.7 ± 8.2; WKY = 103.7 ± 6.9; *p* = 0.007; Figure 3D). We further report a significant difference in DBP between SHRs and WKY rats P30 in wakefulness (SHR = 77.3 ± 8.1; WKY = 63.4 ± 6.8; *p* = 0.009; Figure 3E) and NREM sleep (SHR = 75.5 ± 8.2; WKY = 62.1 ± 6.5; *p* = 0.01; Figure 3F), but only in wakefulness at P25 (SHR = 67.3 ± 9.9; WKY = 54.8 ± 7.0; *p* = 0.047; Figure 3E).

Notably, there were no significant differences in MAP between SHRs and WKY rats in wakefulness at P15 or P20 or NREM sleep at P15 or P20. This persisted for SBP and DBP except for DBP in NREM sleep, where there were no significant differences at P15, P20, or P25.

### 3.4 Effects of eliminating excess OX neurons on MAP and ventilatory chemoreflex in SHRs

**FIGURE 4:**
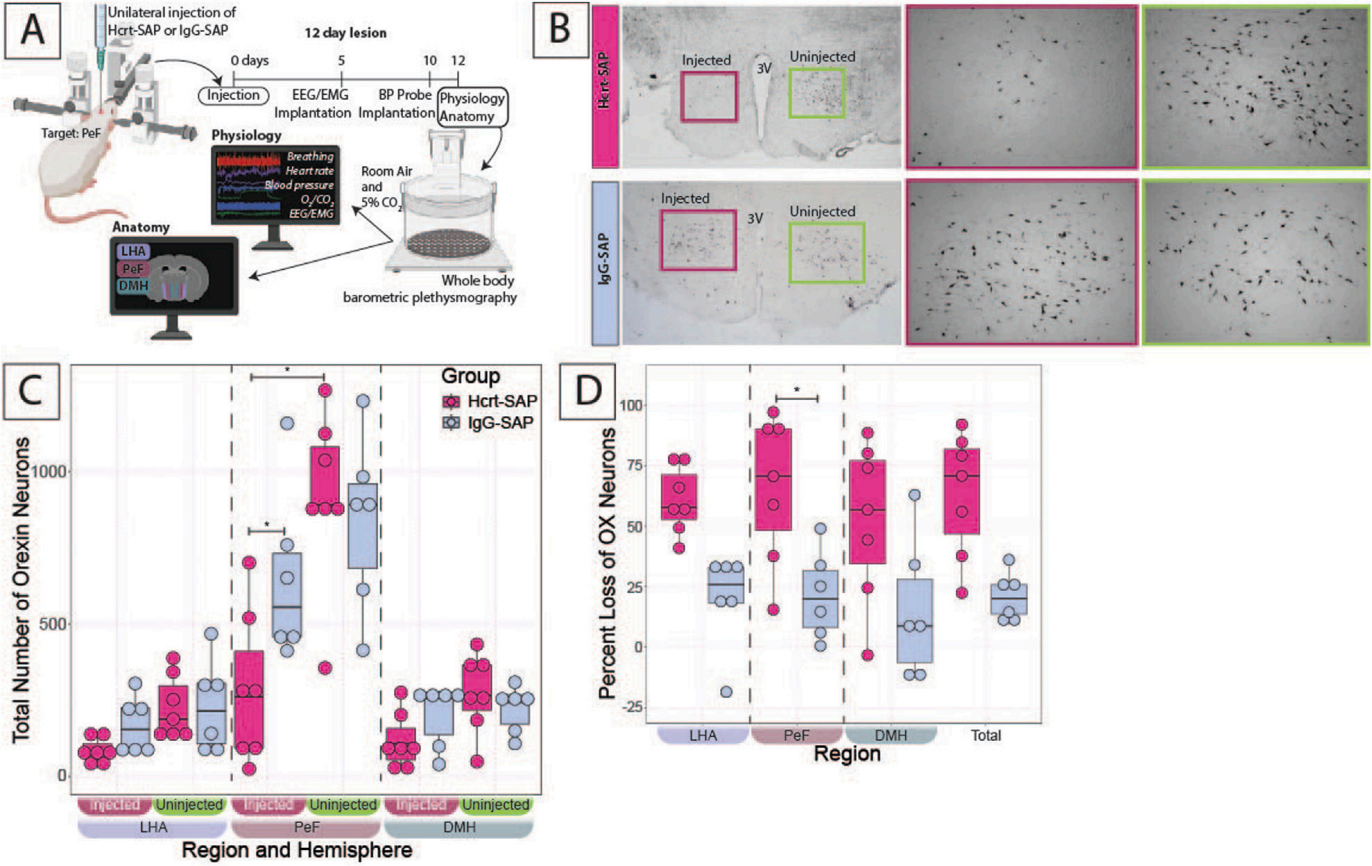
Verification of Hcrt–SAP effect on eliminating excess OX neurons in SHRs. **(A)** Schematic showing the experimental paradigm for these experiments including the physiological outcomes shown in Figure 5. Representative images **(B)** left panel show a full view of the hypothalamus (10X) of Hcrt–SAP injected (left) and non–injected (right) hemisphere, and lower panels show higher and right panel ×20 magnification photomicrographs of the areas encompassed by the squares of the left panel, where Hcrt–SAP–injected lesion is shown in the top and IgG–SAP–injected control is shown in the bottom. **(C)** Shows a comparison of the total number of orexin neurons in Hcrt–SAP vs. IgG–SAP injected SHRs separated by hemisphere (injected vs. uninjected). **(D)** Shows the percent loss of OX neurons in the injected hemisphere in each region and the total hypothalamus as calculated using Equation 1. *Statistically significant difference as indicated with a linear mixed effects model and a Tukey HSD post–hoc test. Abbreviations: Hcrt–SAP, hypocretin–2–saporin; IgG–SAP, IgG–saporin; OX, orexin–A; DMH, dorsomedial hypothalamus; PeF, perifornical zone; LHA, lateral hypothalamic area. Data are shown as mean ± standard deviation.

**FIGURE 5:**
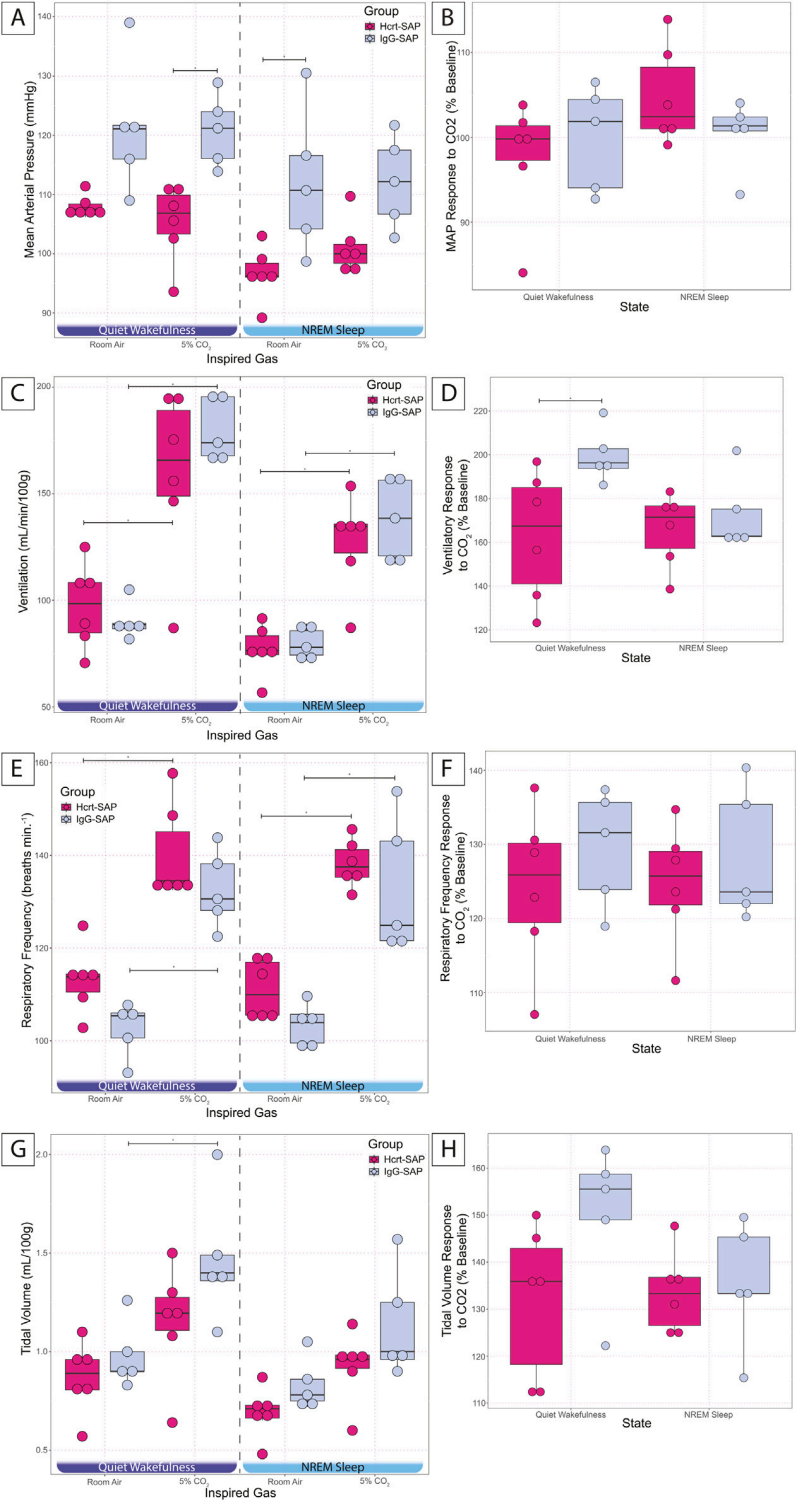
Effects of elimination of excess OX neurons on MAP and CO_2_ chemoreflex in SHRs. MAP in Hcrt–SAP and IgG–SAP injected SHRs **(A)** in quiet wakefulness and NREM sleep during room air and 5% CO_2_ exposure and **(B)** the percent of baseline MAP response to hypercapnia in quiet wakefulness and NREM sleep are shown. **(C)** Shows ventilation in room air and 5% CO_2_ in quiet wakefulness and NREM sleep and **(D)** shows the percent of baseline ventilatory response to hypercapnia. **(E)** Represents respiratory frequency and **(G)** tidal volume in quiet wakefulness and NREM sleep during room air and 5% CO2 exposure and the percent of baseline responses to hypercapnia for **(F)** respiratory frequency and **(H)** tidal volume. *Indicates significant differences as determined with a linear mixed effects model and a Tukey HSD post–hoc test. Abbreviations: NREM, non–rapid eye movement sleep; Ctr, controls; Hcrt–SAP, hypocretin–2–saporin; IgG–SAP, IgG–saporin; VE, ventilation; MAP, mean arterial blood pressure; RA, room air. Data are shown as mean ± standard deviation.

To determine whether excess OX activity is necessary for developing hypertension during development, we evaluated the effects of eliminating some OX–neurons via Hcrt–SAP injection into the hypothalamus on MAP and ventilatory hypercapnic chemoreflex in SHRs during a developmental period (Figures 4, 5).

Table 2 records the ages and body weights at the time of injection and the physiology experiment. There were no significant differences in these parameters between the two groups. Physiology experiments were completed exactly 12 days post–lesion for each animal, with injection ages ranging from P25 to P28 and experimental ages ranging from P37 to P40.

Figure 4A shows the experimental protocol for the SHRs, where the starting age at 0 days is P25–28 and the age at physiology at 12 days is P37–40. Representative images of Hcrt–SAP injected and uninjected hemispheres, as well as IgG–SAP injected and uninjected hemispheres in an SHR, are shown in Figure 4B, and the quantified effect of Hcrt–SAP on the number of OX neurons is shown in Figures 4C,D.

To determine the number of remaining OX neurons in the hypothalamus, the number of OX neurons was first compared between the injected and uninjected hemispheres in the same SHR (Figure 4C). In IgG-SAP injected control SHRs, there was a minimal decrease in the number of OX neurons in the injected hemisphere relative to the uninjected hemisphere, mostly notable in the PeF. However, the loss was not statistically significant and was likely due to mechanical injury during the injection procedure (Figure 4B bottom panel). While in Hcrt–SAP injected SHRs, there was a significant decrease in the number of OX neurons in the PeF resulting from the Hcrt–SAP lesion in the injected hemisphere vs. uninjected hemisphere (injected = 284.9 ± 248.6; uninjected = 918.3 ± 288.1; *p* = 6.13 × 10^−7^). There was also a significant difference in the number of OX neurons in the injected hemisphere when comparing Hcrt–SAP and IgG–SAP in the PeF (Hcrt = 284.9 ± 248.6; IgG = 649.2 ± 283.1; *p* = 0.03).

Cell loss was then compared between Hcrt–SAP and IgG–SAP injected SHRs using the percentage loss of OX neurons as calculated using Equation 1 (Figure 4D). In the injected hemisphere, Hcrt–SAP injected SHRs lost significantly more OX neurons than IgG–SAP injected control SHRs in the PeF (Hcrt = 65.8% ± 30.5; IgG = 21.5% ± 18.2; *p* = 0.04). We also see a trend in the total percent OX neuron loss (Hcrt = 63.3% ± 25.8; IgG = 20.8% ± 10.2; *p* = 0.05). Additionally, we can estimate the total overall loss of OX neurons in the unilateral injection paradigm in the whole brain based on the assumption that both hemispheres would have an equal number of OX neurons if there were no injections. Thus, the total percentage loss of OX neurons in the whole brain would equal roughly ½ of the percent loss of OX in the injected hemisphere described in Figure 4D. In Hcrt–SAP injected SHRs, the estimated total percent loss of OX neurons in the brain would be 31.6%, which is half of the percent loss in the injected hemisphere (63.3%) *versus* 10.4% in IgG–SAP control SHRs. This is a rough estimation as we cannot know how many OX neurons existed in injected animals before injection. So, this is our proxy to estimate the total loss overall. This is a crucial estimation as previous studies have shown that SHRs have ∼30% more OX neurons than age-matched normotensive WKY rats. Therefore, the reduction of OX neurons in SHRs reached in our experiments would roughly bring OX neuron levels to that of control WKY rat levels. However, the loss in our experiment is not evenly distributed across both hemispheres.

The effects of elimination of excess OX neurons on the cardiorespiratory response to hypercapnic challenge are shown in Figure 5. All data are from the dark period because OX is most active during this period and was expected to show the largest measurable outcome. At baseline in room air, Hcrt–SAP lesioned SHRs showed a trend for reduced MAP compared to IgG–SAP control SHRs in quiet wakefulness (Hcrt = 107.9 ± 1.9; IgG = 121.4 ± 11.1; *p* = 0.07) and a significant reduction in NREM sleep (Hcrt = 96.6 ± 4.5; IgG = 112.1 ± 12.3; *p* = 0.02). We saw no significant differences in tidal volume, respiratory frequency, or ventilation in room air during quiet wakefulness and NREM sleep.

In 5% CO_2_, there is a significant reduction in MAP for Hcrt–SAP treated SHRs in quiet wakefulness (Hcrt = 105.3 ± 6.6; IgG = 120.8 ± 6.0; *p* = 0.02; Figure 5A), but no change in NREM sleep. However, we saw no significant increase in MAP in response to 5% CO_2_ for either group. We did note a significant increase in ventilation in response to 5% CO_2_ for both Hcrt–SAP SHRs and IgG–SAP SHRs during quiet wakefulness [Hcrt (Room air = 97.4 ± 19.9; 5% CO_2_ = 159.0 ± 40.4; *p* = 0.0003); IgG (Room air = 90.2 ± 8.7; 5% CO_2_ = 179.7 ± 14.7; *p* = 2.42 × 10^−6^)] and NREM sleep [Hcrt (Room air = 76.8 ± 11.9; 5% CO_2_ = 127.4 ± 22.7; *p* = 0.005); IgG (Room air = 79.8 ± 7.4; 5% CO_2_ = 138.0 ± 19.1; *p* = 0.003)](Figure 5C). We only saw a significant increase in tidal volume in response to 5% CO_2_ in IgG–SAP SHRs in quiet wakefulness (Room air = 0.98 ± 0.17; 5% CO_2_ = 1.47 ± 0.33; *p* = 0.02; Figure 5G). However, we did see a significant increase in respiratory frequency in response to 5% CO_2_ for both Hcrt–SAP SHRs and IgG–SAP SHRs during quiet wakefulness [Hcrt (Room air = 113.2 ± 7.2; 5% CO_2_ = 140.2 ± 10.4; *p* = 5.76 × 10^−5^); IgG (Room air = 102.6 ± 5.9; 5% CO_2_ = 132.6 ± 8.4; *p* = 4.40 × 10^−5^)], and NREM sleep [Hcrt (Room air = 111.1 ± 6.3; 5% CO_2_ = 138.2 ± 5.1; *p* = 5.41 × 10^−5^); IgG (Room air = 103.4 ± 4.6; 5% CO_2_ = 133.0 ± 14.7; *p* = 6.01 × 10^−5^)](Figure 5E), which is likely the driver for the increase in ventilation noted in both groups in each state (Figure 5C). Overall, when analyzing the hypercapnic response, we did not see a change in the percent MAP hypercapnic response compared to baseline during quiet wakefulness. There was a pattern for a reduced respiratory frequency (Figure 5F) and tidal volume (Figure 5H) in response to 5% CO_2_ in quiet wakefulness. While these were not significant differences, they created a combinatorial effect of a significant reduction in the ventilatory response to 5% CO_2_ in Hcrt–SAP SHRs compared to IgG–SAP SHRs in quiet wakefulness (Hcrt = 163.0% ± 29.4; IgG = 199.7% ± 12.4; *p* = 0.04; Figure 5D). No changes were seen in any hypercapnic chemoreflex as a percent baseline in NREM sleep.

**FIGURE 6:**
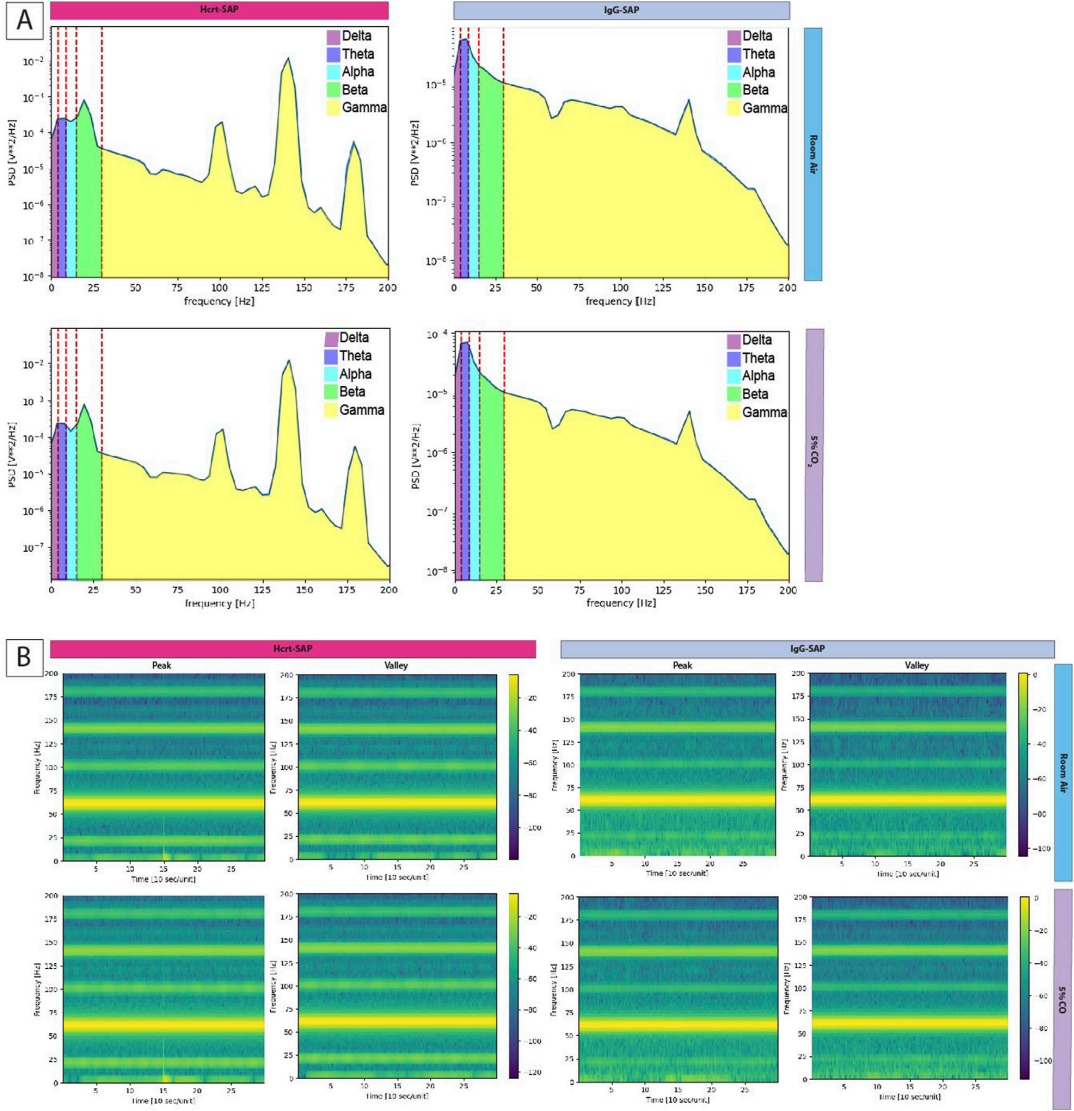
Frequency distribution of EEG following OX elimination in SHRs. **(A)**Welch periodograms of EEG signal frequencies of two representative rats, one treated with Hcrt–SAP (left) and one with IgG–SAP (right) during room air (top row) and 5% CO_2_ (bottom row). Each Power Spectral Density (PSD) band is labeled by color showing the spectral range included in each band where delta (0–4 Hz) is purple, theta (four to eight Hz) is dark blue, alpha (8–15 Hz) is light blue, beta (15–30 Hz) is green, and gamma (30–200 Hz) is yellow. **(B)** Time series spectrograms showing EEG frequency prevalence 2.5 min before and after a peak in EMG signal and 5 min during quiet EMG (valley) for each group in both room air and 5% CO_2_. Abbreviations: Hcrt–SAP, hypocretin–2–saporin; IgG–SAP, IgG–saporin; Hz, Hertz; PSD, Power Spectral Density.

**FIGURE 7:**
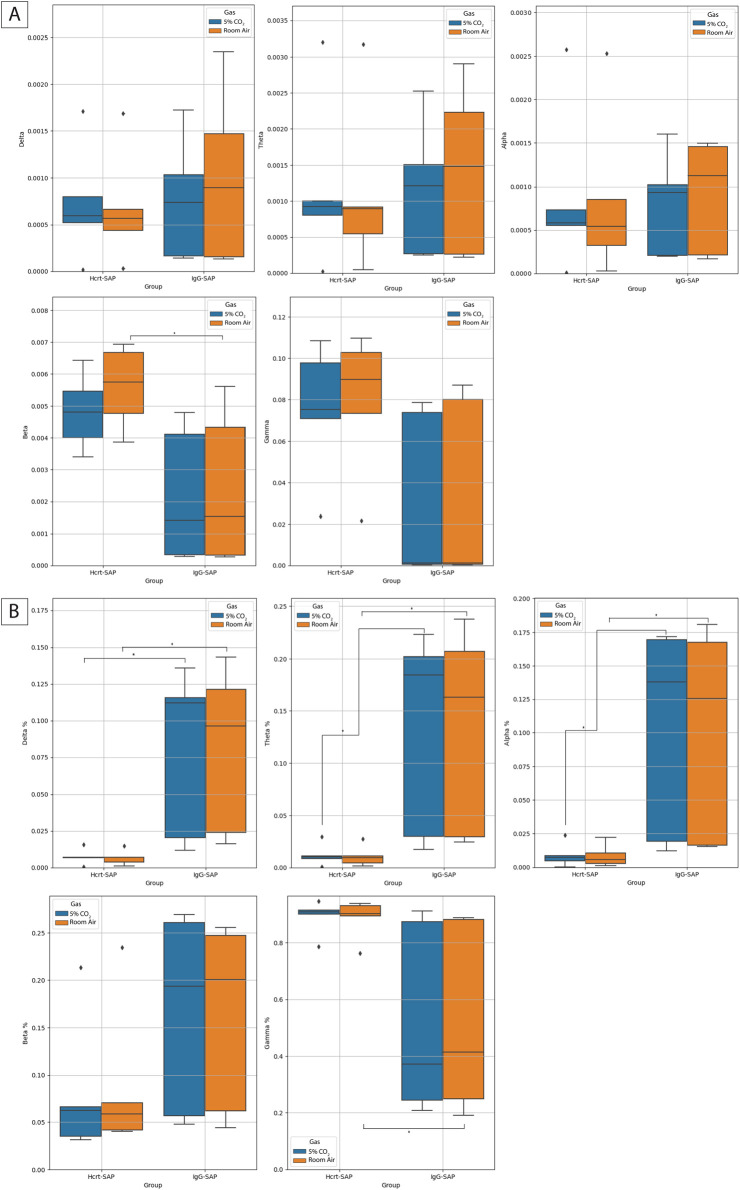
Effects of eliminating excess OX neurons on normalized EEG frequency distributions. **(A)** Box plots indicating the normalized band value averages for Hcrt–SAP and IgG–SAP animals in room air (orange) and 5% CO_2_ (blue) in each of the spectral bands. The percent indicates the percent of PSD comprised of the given frequency band. Diamonds indicate possible outliers. The middle line on the boxplot is the average and error bars show the standard deviation. **(B)** Box plots indicating the raw band value averages for Hcrt–SAP and IgG–SAP animals in room air (orange) and 5% CO_2_ (blue) in each of the spectral bands. Diamonds indicate possible outliers. The middle line on the boxplot is the average and error bars show the standard deviation. *Indicates significant differences as determined with a Kruskal Wallis test. *Indicates significant differences as determined with a Kruskal Wallis test. Abbreviations: Hcrt–SAP, hypocretin–2–saporin; IgG–SAP, IgG–saporin.

For state changes, representative Welch’s periodograms for Hcrt–SAP and IgG–SAP in both room air and 5% CO_2_ are shown in Figure 6A. While we see no appreciable differences between gas exposures, there are qualitative differences in the distribution of the EEG frequencies between Hcrt–SAP and IgG–SAP groups. To examine further state differences between the gas exposure conditions, we created a time series of spectrograms showing frequencies from 2.5 min before and 2.5 min after a peak in the EMG signal or a quiet period (valley) in the EMG signal for each group during room air and 5% CO_2_ (Figure 6B). While additional noise and slight suppression of higher frequency bands during 5% CO2 exposure are noted, there are no fundamental differences in distribution by gas condition. However, you can see an appreciable change in lower frequency bands in the Hcrt-SAP group at the middle time point that is absent in the IgG-SAP group. These qualitative patterns are quantified as the raw Power Spectral Density (PSD) bands in each frequency group (delta (0–4 Hz), theta (four to eight Hz), alpha (8–15 Hz), beta (15–30 Hz), and gamma (30–200 Hz) (Figure 7A). There were no significant differences by gas type across any of the PSD bands. There was a significant increase in the beta band between Hcrt–SAP and IgG–SAP during room air (*p* = 0.0472), but not in 5% CO_2_ (*p* = 0.75). There was also a significant difference between Hcrt–SAP and IgG–SAP groups in the gamma band when data were combined (*p* = 0.01911). In the normalized data, there were significant differences between the lower frequency bands (delta, theta, and alpha) during both room air and 5% CO_2_ [(delta: *p* = 0.00902 RA, *p* = 0.0163 CO_2_) (theta: *p* = 0.0163 RA, *p* = 0.0163 CO_2_) (alpha: *p* = 0.0283 RA, *p* = 0.0283 CO_2_)](Figure 7B). We also saw a significant increase in the gamma band between Hcrt–SAP and IgG–SAP during room air (*p* = 0.0472), but not 5% CO_2_. The IgG–SAP group showed greater variability in all PSD bands as seen in Figure 7.

## 4 Discussion

An overactive OX system has been linked to neurogenic hypertension in adult SHRs (Lee et al., 2013; Li et al., 2013; Li et al., 2016). Our key findings are:

1. SHRs have significantly more OX neurons than WKY rats by P15 before a measurable difference in MAP at ∼ P25.
2. The increase in OX neurons is likely due to exaggerated neurogenesis during postnatal development rather than the maturation of existing neurons.
3. Reducing the number of OX neurons prevents the rise in MAP and the heightened chemoreflex, which indicates that these neurons contribute to the development of neurogenic hypertension in the SHR model.

### 4.1 Postnatal development of excess OX neurons via increased OX neurogenesis in SHRs

In normotensive animals, mRNA expression of OX begins around E12–18 with progressively increasing levels of mRNA and protein expression during postnatal development until about P30, wherein levels remain stable into adulthood (Yamamoto et al., 2000; Steininger et al., 2004; Stoyanova et al., 2010; Iwasa et al., 2015; Sawai et al., 2010; Amiot et al., 2005; den Pol et al., 2001; Ogawa et al., 2017). In adult hypertensive rodents, SHRs and hypertensive mice (BPH/2J) have about 30% and 20% more OX–ir neurons, respectively, than their age–matched normotensive controls (Jackson et al., 2016; Clifford et al., 2015). We have previously reported this increase can be observed in a pre–hypertension period at P30–58 ^22^. However, it was not clear whether SHRs were born with excess OX neurons or if the excess population developed postnatally, and if the increase was due to later OX marker expression (maturation) or increased neurogenesis in postnatal development.

In this study, we found that SHRs and normotensive WKY rats had similar numbers of OX neurons at P7–8; however, by P15, SHRs had significantly more OX neurons than age-matched normotensive controls (Figure 1). These data suggest that SHR pups experienced an exaggerated surge in the total number of OX neurons postnatally between P7 and P16, which BrdU labeling indicates is driven by newly born neurons that express OX rather than embryonically born neurons expressing OX later in development. However, this does not eliminate the possibility that some of the difference could be driven by a difference in the number of prenatally born neurons that has not reached statistical significance in our study. BrdU is commonly used to identify newly proliferating cells in the brain (Menyhárt et al., 2016; Miller and Nowakowski, 1988; Cameron and Mckay, 2001), and multiple 50 mg kg^–1^ injections (i.p.) of BrdU can specifically and sufficiently label newly generated neurons (Miller and Nowakowski, 1988; Cameron and Mckay, 2001; Takahashi et al., 1992; Wojtowicz and Kee, 2006). While there are several caveats with BrdU as a marker of neurogenesis, including the innate toxicity of BrdU and the possibility of labeling other changes in DNA structure, our WKY control groups experienced the same treatment as the SHR study group. Furthermore, the mutagenic properties of BrdU are not a concern due to the acute nature of our experiments. Additionally, the increased number of OX-positive neurons at later time points strongly suggests that the number of neurons marked due to DNA repair or cell death mechanisms, if any, is exponentially smaller than the number of neurons marked for true neurogenesis.

Compared to age-matched normotensive WKY pups, SHR pups had significantly more newly proliferated OX neurons, which were positive for both BrdU and OXA (BrdU/OX–ir), in the hypothalamus at P5–9 (Figure 2E). Importantly, because of the experimental paradigm, this age group also likely includes neurons born within the P10–14 group. Therefore, the increased neurogenesis may represent a larger developmental period than solely P5–9. SHR pups had ∼1,300 more OX–ir neurons in the hypothalamus than age-matched WKY rat pups at P15. Of all neurons counted at P15–16 in SHRs (∼3,700), it is estimated that ∼44% (∼1,600) were contributed by newly proliferated BrdU/OX–ir neurons from combined P5–9 and P10–14 neurogenesis and the remaining 54% were likely contributed by OX–ir neurons born before P5 that matured during the same period. In WKY rats, we only measured ∼2,400 neurons at the same age, wherein ∼32% (∼770) were contributed by neurogenesis from P5–14. It is also important to emphasize that these newly proliferated OX neurons are expected to be functional, as evidenced by their production of OX neuropeptide identified via positive immunohistochemical staining for orexin–A, similar to a previous report in 8–weeks–old rats (Xu et al., 2005). Importantly, there is only a ∼10% difference in newly generated neurons at P15–16 when we see a significant difference between SHR and WKY rat pups. This indicates that neurogenesis of OX neurons is a natural maturation of this system. Still, it does suggest there may be other factors before this period contributing to increased OX neurons in SHRs. However, the number of OX neurons derived from neurogenesis in SHRs (∼1,600) is greater than the total difference between SHR and WKY rats (∼1,300), which indicates that despite comparable percentage contributions of neurogenesis at P5–14, the number derived from neurogenesis in SHRs is much greater and probably responsible for the significant difference in neurons at that age.

Emerging evidence shows that postnatal and adult neurogenesis are present in the hypothalamus (Xu et al., 2005; Kokoeva et al., 2005; Rojczyk–Gołȩbiewska et al., 2014; Sousa–Ferreira et al., 2014), and α–tanycytes are likely the neural progenitor cells for generating peptidergic neurons, e.g., OX neurons, in the hypothalamus (Xu et al., 2005; Lee, 2012; Rizzoti and Lovell–Badge, 2017). Using BrdU, Amiot et al. demonstrated that most OX neurons are generated between embryonic days 11 and 14 ^28^, while others showed that OX neurogenesis in the hypothalamus persists post–E14 and into adulthood (Chang et al., 2013; Chang et al., 2012). Xu et al. further showed that hypothalamic BrdU–identified neurogenesis persists into postnatal 8 weeks of age, and they determined that some of the BrdU–positive neurons expressed OX (Xu et al., 2005). Thus, our findings are aligned and consistent with previous reports of thalamic postnatal neurogenesis, offering additional confirmatory evidence of this relatively new phenomenon. Notably, our work describes a time course of SHR OX neurogenesis shortly (∼10 days) preceding the development of a higher MAP in SHRs that provides a plausible cellular mechanistic explanation for SHR hypertension (see discussion below).

It is currently unknown what may cause this increase in OX neurogenesis; however, we can speculate that genetic predisposition combined with early postnatal triggers may play a role. Many studies have identified single nucleotide polymorphisms that, when taken together, significantly contribute to hypertension in rats and humans (Pravenec and Kurtz, 2010; Natekar et al., 2014). McCarty and Lee further showed that SHR pups that were cross-fostered by WKY dams had significantly lower MAP than SHRs reared by their own mother. On the other hand, WKY pups that were cross-fostered by an SHR dam had no change in their MAP (McCarty and Lee, 1996), suggesting a contributing maternal care component that only manifests on the SHR background. These data suggest that while the genetic factors are essential to the development of higher MAP in postnatal development, some aspect of the postnatal development of SHRs is likely stress-related and serves as a contributing factor that is also required for the full development of hypertension. SHR dams have been shown to have increased stress levels, increased tactile stimulation of the pups, altered nutrient exchange during lactation, excessive grooming, and restlessness–all of which could be or contribute to this secondary trigger (McCarty and Kopin, 1978; Tucker and Johnson, 1981; Myers et al., 1989; McCarty et al., 1992; Krukoff et al., 1999). Additionally, chronic stress, e.g., foot–shock, can induce hypertension and double the number of OX neurons in the hypothalamus in normotensive rats (Xiao et al., 2013). It is possible that a polygenic predisposition accompanied by prenatal and/or postnatal exposures to increased levels of stress produces an additive effect that leads to the exaggerated OX neurogenesis. The OX system is associated with hyperarousal, anxiety/stress, and autonomic functions (Huang et al., 2010; Kayaba et al., 2003; Furlong et al., 2009; Johnson et al., 2010; Nattie and Li, 2010), and this early surge of OX activity in SHRs may produce an aberrant excitatory drive to many cardiovascular-related nuclei in the brainstem and spinal cord, resulting in the facilitation of the pathological development of hypertension in SHRs.

### 4.2 Relationship between surge in OX activity and MAP during development in SHRs

The chronological relationship between increased OX activity and MAP in SHRs during development was largely unknown. It is known that MAP increases progressively and rapidly from newborn (∼15–25 mmHg) through the first 3 weeks of life (∼80–90 mmHg) in normotensive rats (Zicha and Kunes, 1999). Similar to these previous reports, in this study, we have found that SHR pups have comparable MAP, diastolic blood pressure (DBP), and systolic blood pressure (SBP) to the background normotensive WKY rat pups during the first 2 weeks of life (Figure 3) (Li et al., 2013; Zicha and Kunes, 1999; Sei et al., 2002; Nagai et al., 2003; Friberg et al., 1989; Dickhout and Lee, 1998). Around 3–4 weeks of age a measurable difference in MAP between SHR and WKY rat pups emerges and the divergence of MAP between the two strains escalates between weeks 4 and 12 (Zicha and Kunes, 1999; Sei et al., 2002; Nagai et al., 2003; Friberg et al., 1989; Dickhout and Lee, 1998; Lais and Brody, 1977). Using a telemetric method in conscious animals, we confirmed that by P25, a small but significant difference in MAP began to emerge between SHR and WKY rat pups in both wakefulness and sleep. As discussed above, in SHRs, the number of OX neurons surges to significantly higher than normotensive controls by P15–16, which is about 10 days prior the emergence of the difference in MAP between SHR and normotensive WKY pups. Even though at this point it is unclear the exact mechanism of such a chronological relationship between increased OX activity and MAP, the closely associated sequential events suggest a potential causal role for the OX system in developing hypertension in SHRs. We speculate that the early surge of OX signaling may provide excess, and necessary, excitatory drive to many cardiorespiratory–related nuclei in the brainstem and sympathetic preganglionic neurons in the spinal cord to increase SNA, which in turn contributs to developing hypertension in SHRs during postnatal development. A causal role for increased OX neurogenesis in SHR hypertension is further supported by our OX cell ablation experiments.

### 4.3 Effects of eliminating excess orexin neurons on MAP and CO_2_ chemoreflex in SHRs

In addition to higher MAP and exaggerated CO_2_ chemoreflex, SHRs also have ∼30% more OX neurons than normotensive control WKY rats at two ages, P30–58 and adult (Li et al., 2016). Here we further showed that excess OX neurons and higher MAP in SHRs emerges in a sequential order during postnatal development at P15 and P25, respectively. If excess OX neurons are necessary for developing and/or maintaining a higher MAP during postnatal development, then elimination of the excess OX neurons in the hypothalamus may be beneficial in preventing or reducing the aberrant increase in MAP during this period. To test this hypothesis, we selectively eliminated some of the OX neurons using Hcrt–SAP in a subset of SHRs from P25–28 and compared their MAP and CO_2_ chemoreflex with IgG–SAP injected SHR controls 12 days post–lesion at P37–40. The result that eliminating ∼30% OX neurons was sufficient to prevent these OX–lesioned SHRs from developing higher MAP and exaggerated CO_2_ chemoreflex during a postnatal developmental period (P37–40; Figures 4, 5), demonstrates that excess OX neurons are required for maintaining increased MAP and exaggerated CO_2_ chemoreflex and aligns with their developmental trajectories, indicating that increased OX neurogenesis is a key component for the development of increased MAP in SHRs.

On average, the lesioned–SHRs without excess OX neurons had a MAP that was ∼14 mmHg lower than non–lesioned SHRs with excess OX neurons (121 vs. 107 mmHg in wakefulness during dark period) at P37–40. The change was similar to that found with OXR blockade in SHRs (Li et al., 2016), where OXR blocker significantly lowered MAP from 121 to 103 mmHg, a level similar to that of WKY rats at the same age (99 mmHg). Although we did not directly compare the MAP of lesioned–SHRs with normotensive WKY rats, our results, combined with previously published reports, suggest that the MAP resultant from excess OX neuron elimination is comparable to the expected MAP of WKY rats at the same age. It should be noted that less conservative statistical approaches were used in the previously cited paper from the lab and that with those less stringent approaches, the difference in MAP during quiet wakefulness also becomes significant in our dataset whereas our more rigorous statistical analysis only shows significance during NREM sleep.

In terms of CO_2_ chemoreflex, we previously showed that SHRs have elevated ventilatory and MAP responses to hypercapnia at young (P30–58) and adult ages and that treating with OXR blocker can normalize such exaggerated CO_2_ chemoreflex in SHRs in wakefulness and sleep. Here, we found that the lesioned–SHRs without excess OX neurons had a significantly lower ventilatory response to hypercapnia than non–lesioned rats with excess OX neurons only in wakefulness (Figure 5D). We speculate that the vigilance state–dependency found here could be contributed by 1) a proven effect of Almorexant to promote sleep, 2) possible developmental differences between OX neurons and OXRs during postnatal developmental period, and 3) methodological differences in OX system modulation, e.g., elimination of OX neurons vs. blockade of OXRs. Eliminating excess OX neurons will result in loss of other neuropeptides that are also produced by OX neurons, e.g., dynorphin (Chou et al., 2001), even though at this point the role of dynorphin on MAP remains unclear. Of course, a long–term study on the effect of eliminating excess OX neurons or OX peptide on MAP and CO_2_ chemoreflex in the future may further provide therapeutic significance in human hypertension.

State changes following decreased OX resulted in decreased low-frequency EEG and increased high-frequency EEG signal (Figures 6, 7). These results suggest that lowering OX to an expected normal level increases wakefulness across room air and 5% CO_2_ exposure. Surprisingly, we did not see a difference in EEG PSD bands between the two gas exposures. This is likely because of the length of the recording analyzed for the EEG analysis wherein a homeostatic steady state would be reached following this level of hypercapnic exposure rather quickly. This may also be of interest for future work as it seems to indicate the effects of a moderate hypercapnic exposure in rodents is not as influential on state as was expected. Perhaps due to the burrowing nature of these animals their tolerance for state changes based on hypercapnia are different than their respiratory and cardiovascular adjustments. Unexpectedly, we saw significant reductions in the normalized PSD bands for the lower frequency bands (delta, theta, and alpha), which would suggest a decrease in sleep states. Lack of OX is the known cause of a subset of narcolepsy and therefore, we would’ve expected an increase in sleep. However, because we are only decreasing the levels to around normal, it is possible that this would not be enough to elicit a pathological sleep response despite the OX activity baseline for SHRs (which is higher than normotensive animals). One possible phenotype captured in this data is the established ADHD characteristics of the SHR (Sagvolden, 2000; Kantak et al., 2008). Although the ADHD and hypertensive phenotypes can be bred apart, which indicates separate genetic drivers, both phenotypes are present in the SHR model. The increase in gamma bands may indicate greater attentive behavior and focus (Guan et al., 2022; Ramlakhan et al., 2020). Although changes in sleep were expected, it may be that the ADHD phenotype complicated the results of the EEG analyses in this particular study. It would be interesting to see these results in SHRs wherein the ADHD phenotypes have been bred out. We would expect to see better sleep consolidation in these cases as SHRs are known to suffer fragmented sleep (Lai et al., 2016).

Together, we report a plausible cellular mechanism for the development of higher MAP and an exaggerated CO_2_ response in SHRs compared to WKY rats using anatomical timelines demonstrating neurogenesis increases before the establishment of increased OX neuron populations in SHRs and ablation of OX neurons eliminating the increased MAP and CO_2_ response. This work significantly contributes to our understanding of OX and provides much–needed evidence to support prior associations. It also paves the way for future work into therapeutic intervention for essential hypertension.

## Data Availability

The original contributions presented in the study are included in the article/supplementary material, further inquiries can be directed to the corresponding author.
